# Supplementary material for: Differences in ligand-induced protein dynamics extracted from an unsupervised deep learning approach correlate with protein–ligand binding affinities
Source: Commun Biol. 2022 May 19;5:481. doi: 10.1038/s42003-022-03416-7 (PMC9120437; doi:10.1038/s42003-022-03416-7)
Supplement: Supplementary file 2 — Supplementary Material [file 42003_2022_3416_MOESM2_ESM.pdf]

Supplementary Material for "Differences in ligand-induced protein dynamics extracted from an unsupervised deep learning approach correlate with protein–ligand binding affinities"

Ikki Yasuda,[1] Katsuhiro Endo,[1] Eiji Yamamoto,[2] Yoshinori Hirano,[1,3] and Kenji Yasuoka*,[1]

[1]*Department of Mechanical Engineering,*
Keio University, Yokohama, Kanagawa, Japan

[2]*Department of System Design Engineering,*
Keio Univsity, Yokohama, Kanagawa, Japan

[3]*Laboratory for Computational Molecular Design,*
RIKEN Center for Biosystems Dynamics Research (BDR), Suita, Osaka, Japan

*Corresponding author. E-mail: yasuoka@mech.keio.ac.jp

I. LOSS FUNCTION FOR DEEP NEURAL NETWORK

Deep neural networks (DNNs) to calculation Wasserstein distance use the loss function with gradient penalty [1],

$$L = \mathbb{E}_{\boldsymbol{s}\sim \boldsymbol{y}_i}\left[f(\boldsymbol{s})\right] - \mathbb{E}_{\boldsymbol{t}\sim \boldsymbol{y}_j}\left[f(\boldsymbol{t})\right] - \mathbb{E}_{\boldsymbol{r}\sim \boldsymbol{R}}\left[f(\boldsymbol{r})(||\nabla_{\boldsymbol{t}} f(\boldsymbol{r})|| - 1)\right]^2 \tag{1}$$

where $\boldsymbol{s}$ and $\boldsymbol{t}$ are short-term trajectories, $\boldsymbol{y}$ is probability distribution of local dynamics ensemble and lower index means system, $\boldsymbol{r}$ is interpolation between $\boldsymbol{s}$ and $\boldsymbol{t}$, i.e. $\boldsymbol{r} = \epsilon\boldsymbol{s} + (1-\epsilon)\boldsymbol{t}$ with ϵ being a uniform random number between 0 and 1. $\boldsymbol{R}$ is probability distribution of $\boldsymbol{r}$.

II. DETAILS OF WASSERSTEIN DISTANCE EMBEDDING

Matrix of Wasserstein distances is embedded into points in a low dimension space. The points are determined to minimize distance loss,

$$DL = \sum_{i<j}(W_{i,j} - ||\boldsymbol{p}_i - \boldsymbol{p}_j||)^2 \tag{2}$$

where W_{ij} is Wasserstein distance in local dynamics ensemble (LDE) distribution of system i and j, $\boldsymbol{p}_i$ is embedded point of system i. The points are optimized in two steps of simulated annealing and gradient descent. Firstly, simulated annealing is performed,

$$\boldsymbol{P}_{new} = \begin{cases} \boldsymbol{P} + \boldsymbol{a}, & \text{if } \exp[(DL - DL_{new})/T] \leq b \\ \boldsymbol{P}, & \text{otherwise} \end{cases} \tag{3}$$

where $\boldsymbol{P}$ is $[\boldsymbol{p_0}, \boldsymbol{p_1}, ..\boldsymbol{p_n}]$ for n systems, $\boldsymbol{a}$ is a vector of uniform random number with the same dimension of $\boldsymbol{P}$, and b is a random number, and T is a variable of the annealing step and determined by,

$$1/T = c\log\left(1 + \frac{s}{S}(-1+e)\right) \tag{4}$$

where c is a constant, s is the number of current step, and S is the maximum number of annealing step, and e is Euler's number. After the simulated annealing, gradient descent was performed,

$$\boldsymbol{P}_{new} = \boldsymbol{P} + \eta\nabla_{\boldsymbol{p}} DL \tag{5}$$

where η is learning rate.

III. ANALYSIS OF FLUCTUATION DYNAMICS OF BRD4 SYSTEMS

The difference in local fluctuation at a local binding site was studied in terms of relaxation dynamics. A positional autocorrelation function [2] $C'_i(\Delta)$ is defined as

$$C'_i(\Delta) = \frac{1}{t-\Delta}\int_0^{t-\Delta} \delta l_i(t')\delta l_i(t'+\Delta)dt' \tag{6}$$

where $\delta l_i(t') = l_i(t') - \langle l_i \rangle$, where l_i is the distance between the center of mass of residue i and the center of geometry of the protein, $\langle l_i \rangle$ is the time average of l_i, and t is the simulation length. We note that only heavy atoms were considered here. The autocorrelation was normalized as follows:

$$C_i(\Delta) = \langle C'_i(\Delta)\rangle / \langle C'_i(0)\rangle \tag{7}$$

Supplementary Fig. 7 shows the relaxation of the 14 residues of BRD4 calculated from molecular dynamics (MD) trajectories in 50–350 ns. Overall, complex systems relaxed faster than the apoprotein system, especially with higher affinity ligands such as ligand (L) 3 and 4. However, the influence of the ligand was specific to the residues. For instance, L1 drove relaxation at Val87 but played an opposite role in Asn140. At the time length used for the LDE (128 ps), there was a variance in the extent of relaxation, implying that the dynamics at this scale differ depending on the system. This suggests that even at 100 ps of dynamics, the ligand-induced dynamics are distinguishable.

IV. EVALUATION OF DYNAMICS STABILITY USING $g(\boldsymbol{x})$

We compared local dynamics of BRD4 systems under different initial condition using $g(\boldsymbol{x})$. Supplementary Fig. 8 shows probability probability distributions of $g(\boldsymbol{x})$ as the reference of L3 system, for three individual MD simulations that consisted of trajectory data for one system. Since L3 was located opposite to the apoprotein in the embedding (Fig. 3b), it was used as a reference here. Overall, the probability distributions were almost identical regardless of the simulations. As an exception, the probability distribution of simulation 2 in the apoprotein system was narrower than the other two simulations, indicating that simulation 2 might be trapped in a local minimum (Supplementary Fig. 8). In addition, a wider range of simulation 2 in L7 corresponds to the ligand dissociation (Supplementary Fig. 6). The result suggests that local dynamics own robustness to initial conditions to a

large extent. As for the shape of the probability distributions depending on the ligand, the apo and L7,10 systems showed wider probability distributions in the direction that enlarges difference from the L3 system, in agreement to the embedding where L7 and L10 systems behaved similarly to the apoprotein. The right-skewed probability distributions in these systems indicate that ligand-unbound-like characteristic dynamics are relatively rare events.

Temporal stability of local dynamics was evaluated using $g(\boldsymbol{x})$. Supplementary Fig. 9 shows $g(\boldsymbol{x})$ as a function of time obtained from simulation 1 of each system. There was no overall rising or falling trend for $g(\boldsymbol{x})$ in systems. Therefore, the local dynamics is speculated in equilibrium.

V. MOLECULAR DYNAMICS SIMULATION LENGTH SUFFICIENT TO COVER LDE

To understand the convergence of Wasserstein distances with respect to the length of MD simulations, Wasserstein distances were calculated using shorter MD simulations and evaluated by the average error ratio E, which is defined as

$$E(t) = \frac{2}{N(N-1)} \sum_{i<j} \left| \frac{W_{i,j}(t) - W_{i,j}(t_{\mathrm{ref}})}{W_{i,j}(t_{\mathrm{ref}})} \right| \tag{8}$$

where t is the time length for simulation, N is the number of systems, i, j are indices for the systems, and t_{ref} is a reference time of 400 ns. The error rate at 400 ns were obtained by running another independent calculation of the reference calculation. Supplementary Fig. 12 shows that the error exponentially decreased with MD simulation length. The simulation length corresponds to the number of data points; this agrees with the fact that the Wasserstein distance calculated from a finite sample has an error that exponentially decreases with the number of samples [3]. The error values were 14, 2.1, 0.42 % in 200, 300, 400 ns, respectively.

VI. SELECTION OF THE LOCAL DYNAMICS TIME LENGTH

To find the optimal LDE time length, the results of embedding from 128 to 12,800 ps were compared (Supplementary Fig. 14). A longer LDE indicates a larger time step between each step for DNN input, conserving the number of data sizes. Overall, the positions of the

systems were largely separated according to the binding energies in any selection. As for the first principal component, while it showed a contrast between apoproteins and holoproteins at 128 ps, the relationship was disturbed with a longer LDE time. In the longer LDE periods, the distances between the systems increased, and the variance in the second principal increased (1,280 ps). Finally, the differences in apoprotein and the two lowest-affinity systems were most emphasized at 6,400 and 12,800 ps, resulting in the principal component deviating from the differences in apoprotein and holoproteins. In addition, there could be a concern that sampling of longer LDEs requires longer MD simulations. Insufficient sampling would lead to more dependence on the initial conditions. Therefore, it was suggested that the LDE time could be selected as short, but sufficiently long for side-chain movements.

[1] I. Gulrajani, F. Ahmed, M. Arjovsky, V. Dumoulin, and A. C. Courville, Improved training of wasserstein gans, in *Advances in neural information processing systems* (2017) pp. 5767–5777.

[2] E. Yamamoto, T. Akimoto, A. Mitsutake, and R. Metzler, Universal relation between instantaneous diffusivity and radius of gyration of proteins in aqueous solution, Physical review letters **126**, 128101 (2021).

[3] P. M. Esfahani and D. Kuhn, Data-driven distributionally robust optimization using the wasserstein metric: Performance guarantees and tractable reformulations, Mathematical Programming **171**, 115 (2018).

[4] C. C. G. ULC, Molecular operating environment (moe), 2020.09 (2020).

[5] M. Aldeghi, A. Heifetz, M. J. Bodkin, S. Knapp, and P. C. Biggin, Accurate calculation of the absolute free energy of binding for drug molecules, Chemical science **7**, 207 (2016).

[6] X. He, S. Liu, T.-S. Lee, B. Ji, V. H. Man, D. M. York, and J. Wang, Fast, accurate, and reliable protocols for routine calculations of protein–ligand binding affinities in drug design projects using amber gpu-ti with ff14sb/gaff, ACS omega **5**, 4611 (2020).

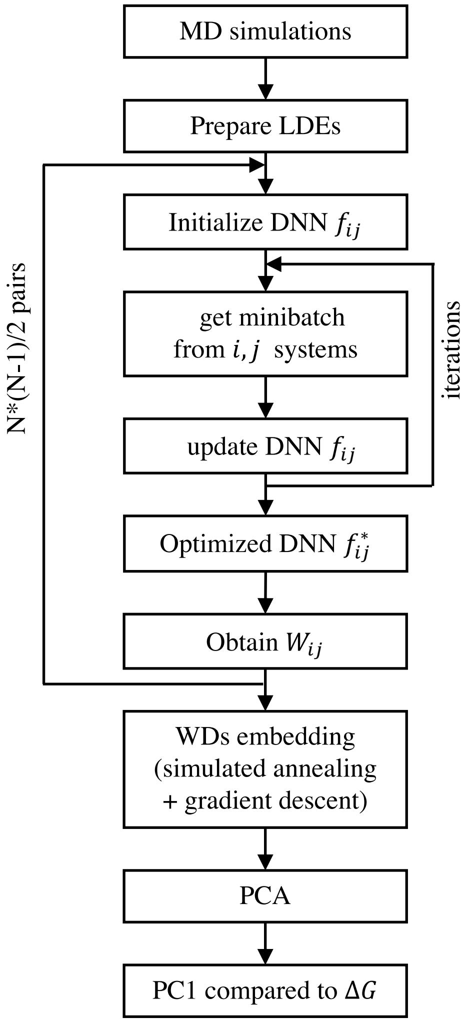

Supplementary Figure 1. Workflow to extract feature of ligand-induced protein dynamics from MD simulations.

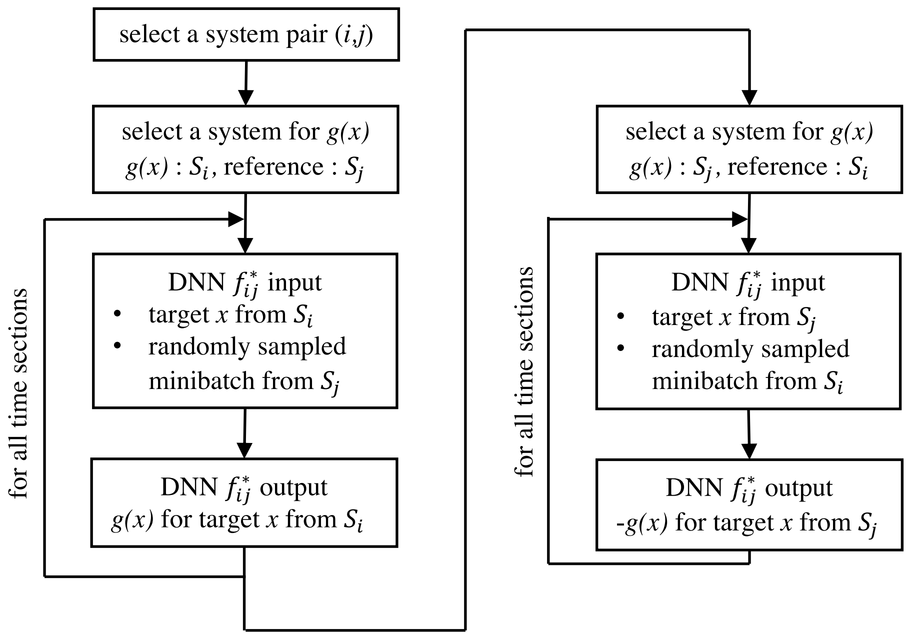

Supplementary Figure 2. Workflow to calculate function $g(\boldsymbol{x})$. S_i means system with ligand i. DNNs are optimized to calculate Wasserstein distances, and used to calculate the function $g(\boldsymbol{x})$.

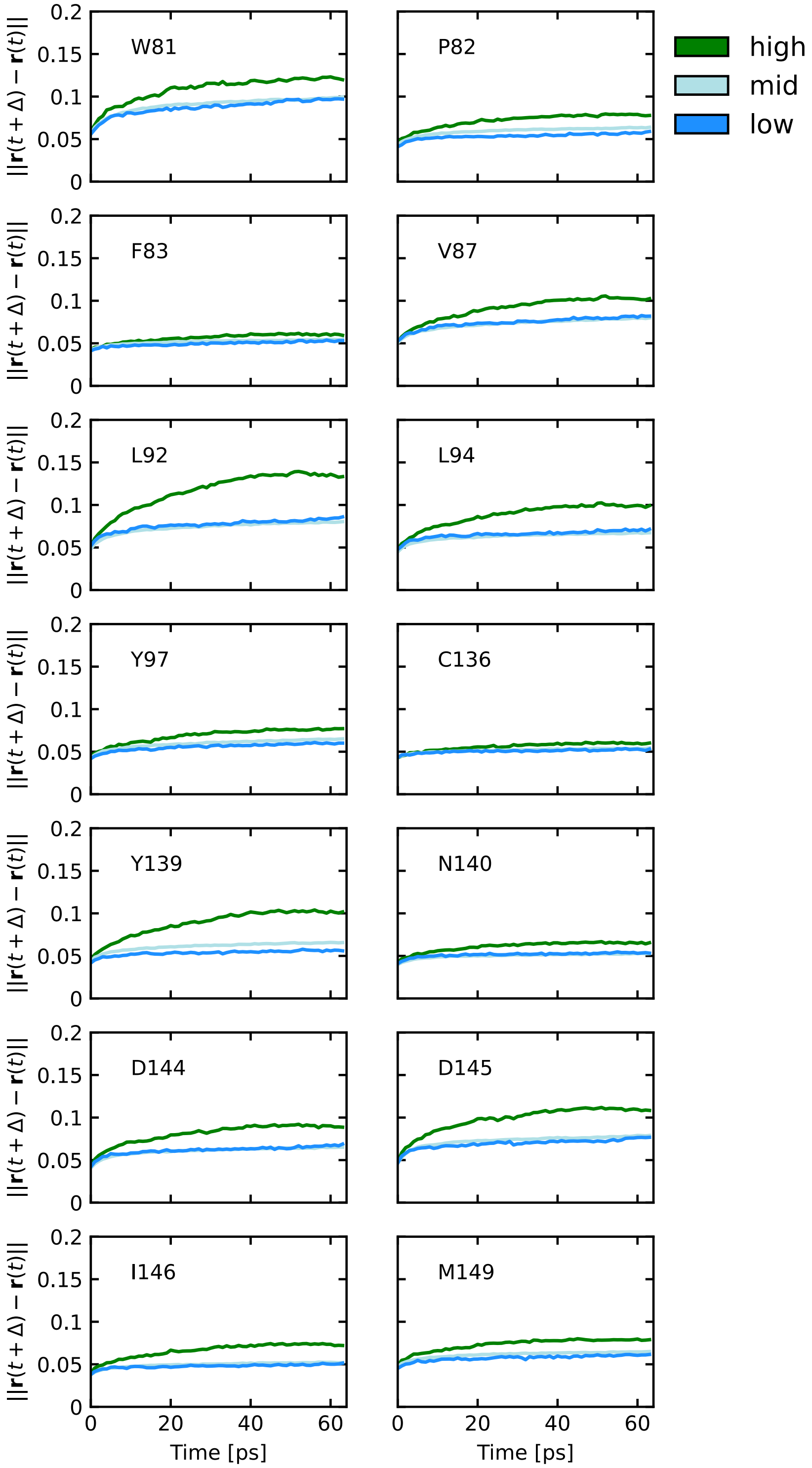

Supplementary Figure 3. Short-term root mean square displacement (RMSD) in BRD4. Short-term trajectories of apoprotien BRD4 are classified to characteristic, non-characteristic or the middle, in comparison to system with ligand 3.

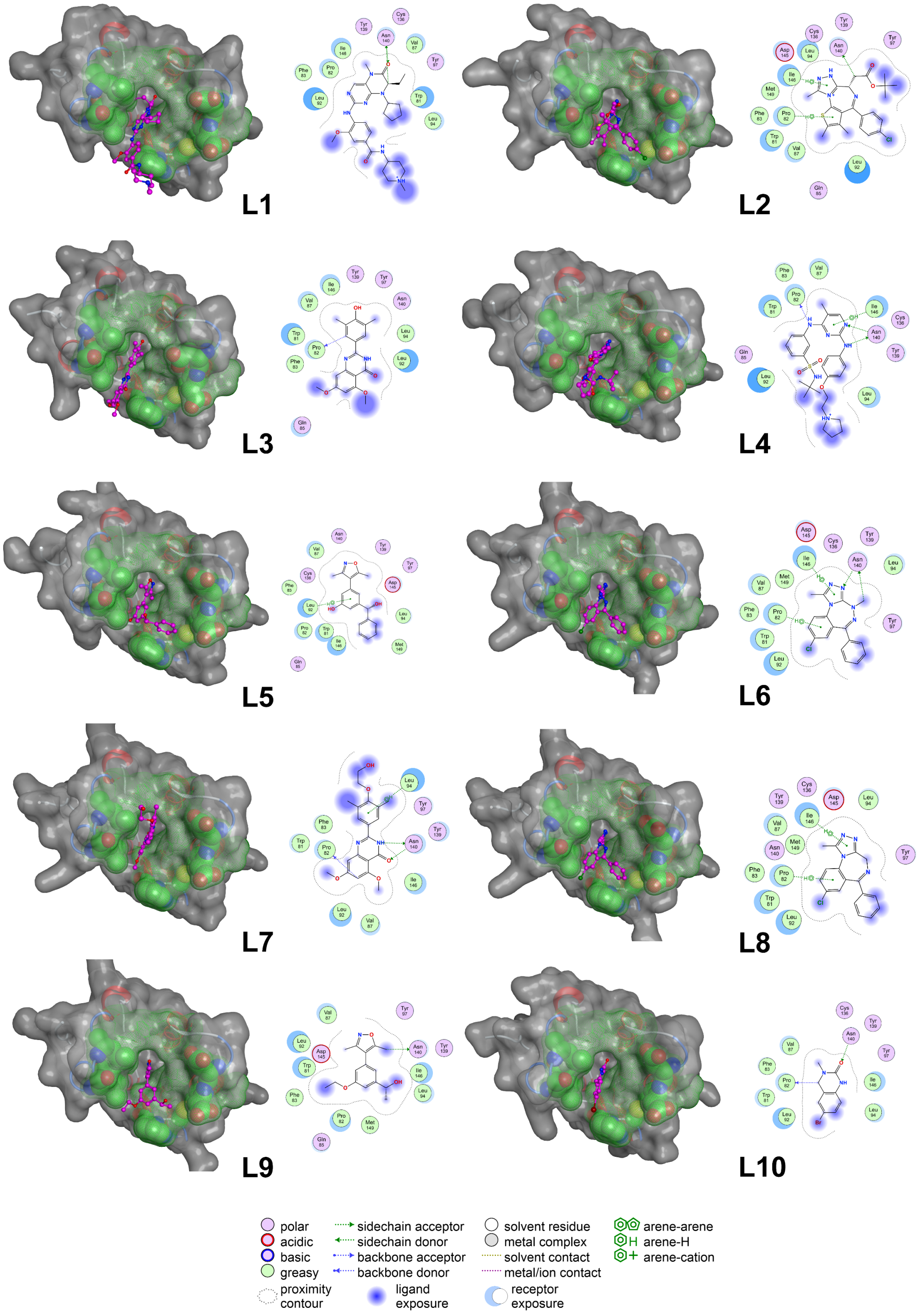

Supplementary Figure 4. BRD4 initial structures and 2D ligand interaction diagrams. Ribbon diagram of the configuration of BRD4 superimposed on the molecular surface. The ligand-binding site, residues in the ligand-binding site, and key residues are shown in green meshed molecular surfaces, pink balls and sticks, and green space-filling with labels, respectively. 2D diagrams are depicted by using the Molecular Operating Environment (MOE) software package [4]

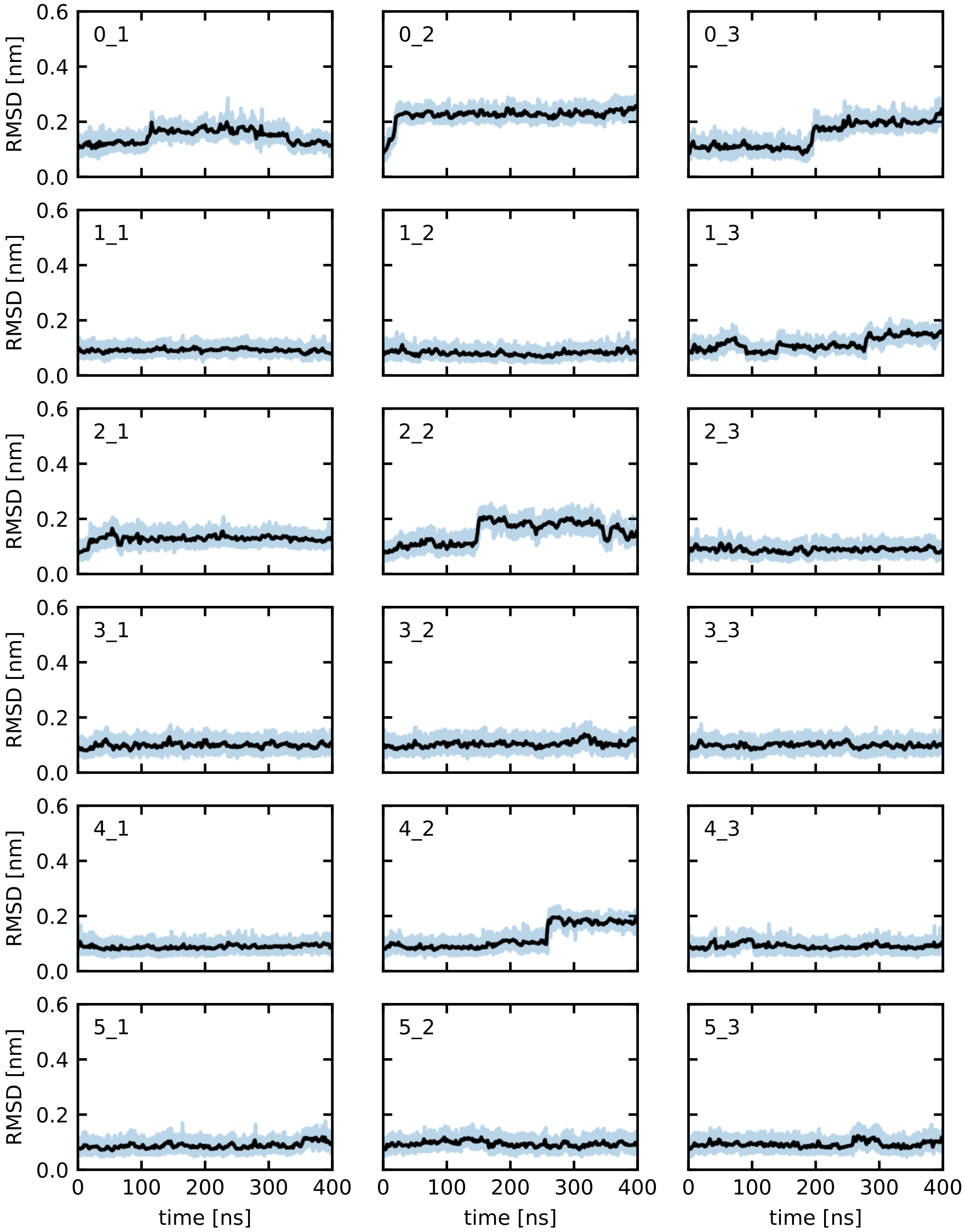
0_1
0_2
0_3
1_1
1_2
1_3
2_1
2_2
2_3
3_1
3_2
3_3
4_1
4_2
4_3
5_1
5_2
5_3
RMSD [nm]
time [ns]
0.0
0.2
0.4
0.6
0
100
200
300
400

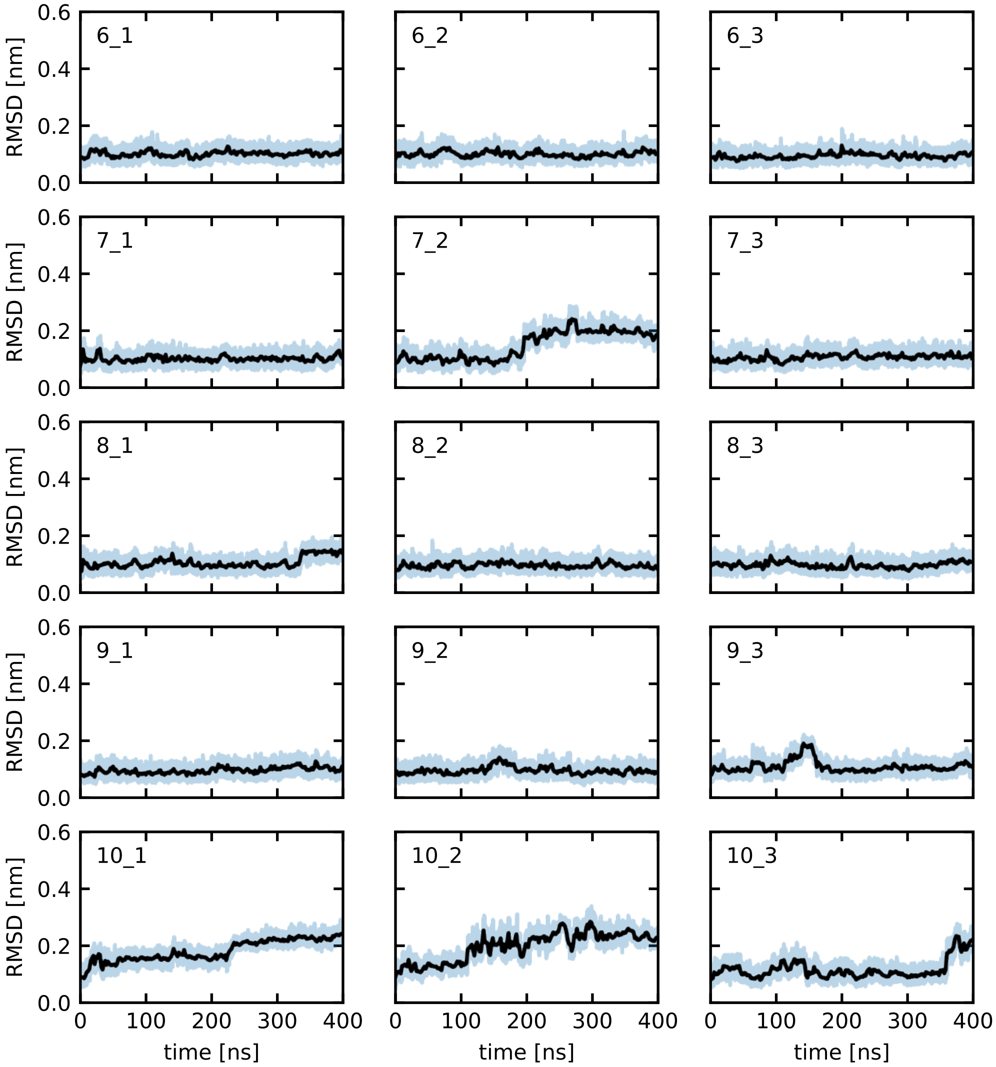

Supplementary Figure 5. Root-mean-square (RMS) deviation of protein in BRD4 systems. CA atoms without terminals were used (59GLN–161ILE). Structured were fitted by the CA atoms. Top-left label indicates system and simulation number. Light blue and black lines show the RMS deviation plotted in every 2 ps and moving average of the RMS deviation in 2 ns, respectively.

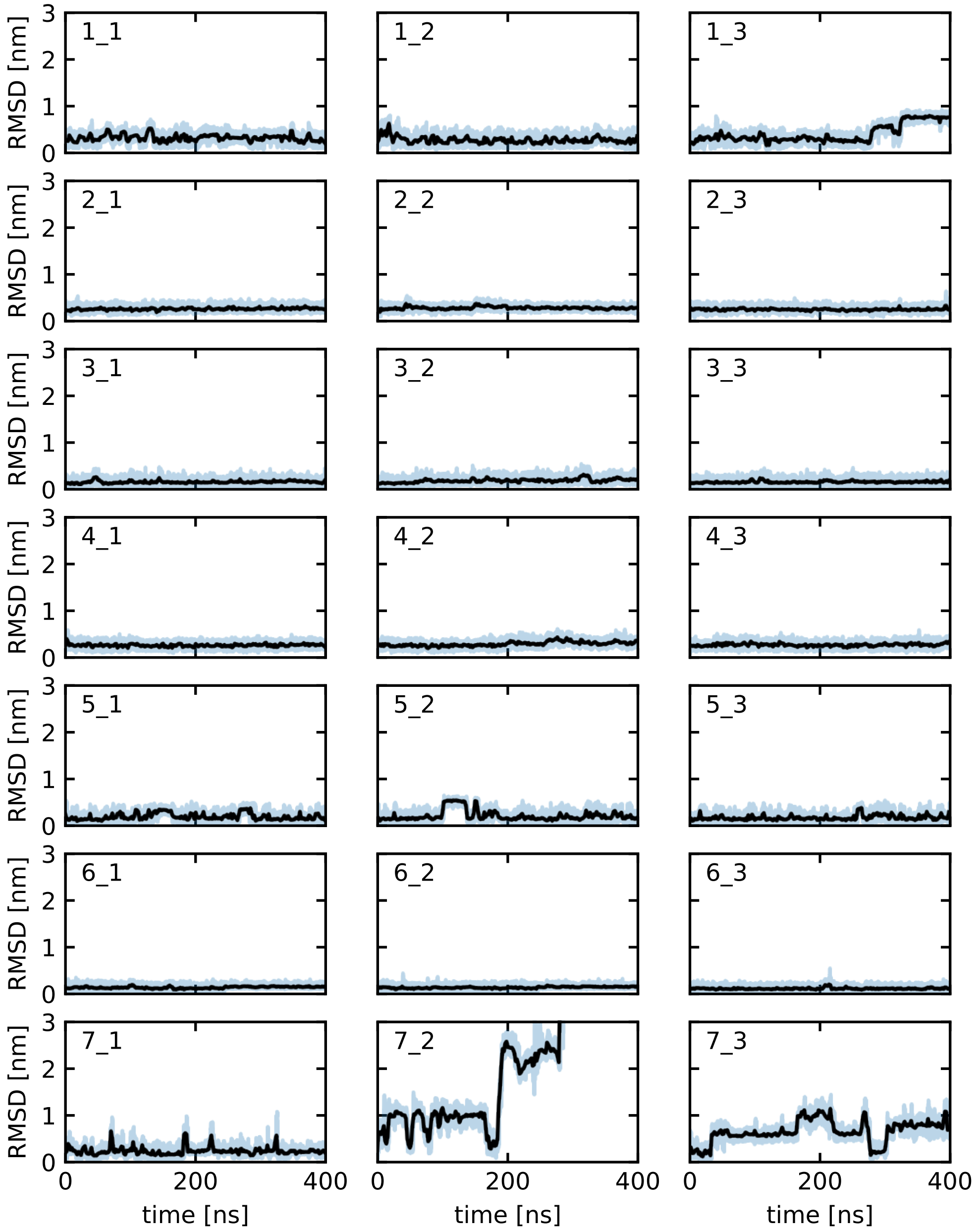
1_1
1_2
1_3
2_1
2_2
2_3
3_1
3_2
3_3
4_1
4_2
4_3
5_1
5_2
5_3
6_1
6_2
6_3
7_1
7_2
7_3
RMSD [nm]
time [ns]
0
200
400
0
1
2
3

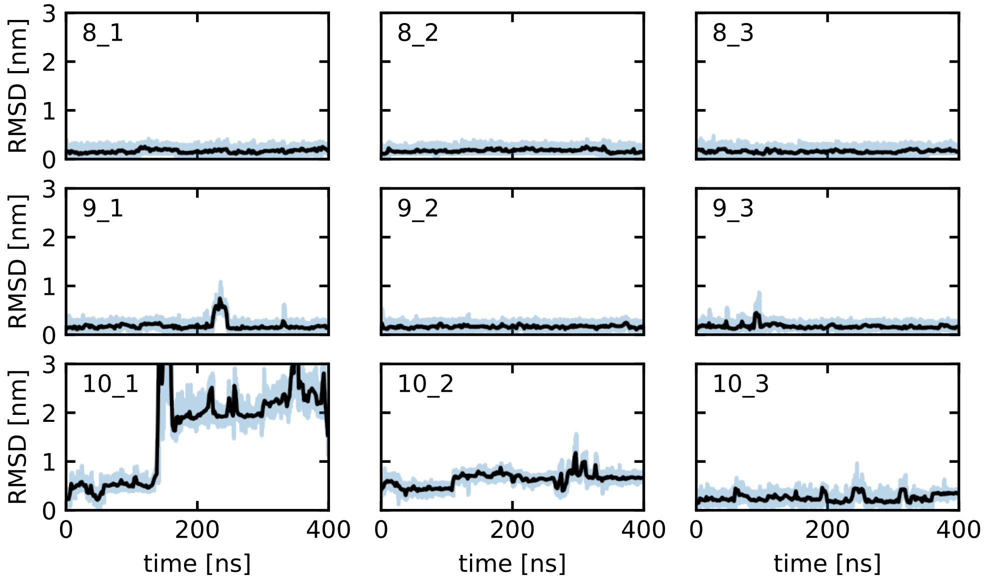

Supplementary Figure 6. RMS deviation of ligand in BRD4 systems. Translation and rotation were removed by fitting by protein CA atoms, and ligand heavy atoms were used to calculate the RMS deviation. Top-left label indicates system and simulation number. Light blue and black lines show the RMS deviation plotted in every 2 ps and moving average of the RMS deviation in 2 ns, respectively. Ligand dissociation was observed in ligand 7 and 10 systems. We assumed that the dissociation reflects lower affinity, and trajectories after the dissociation were still used.

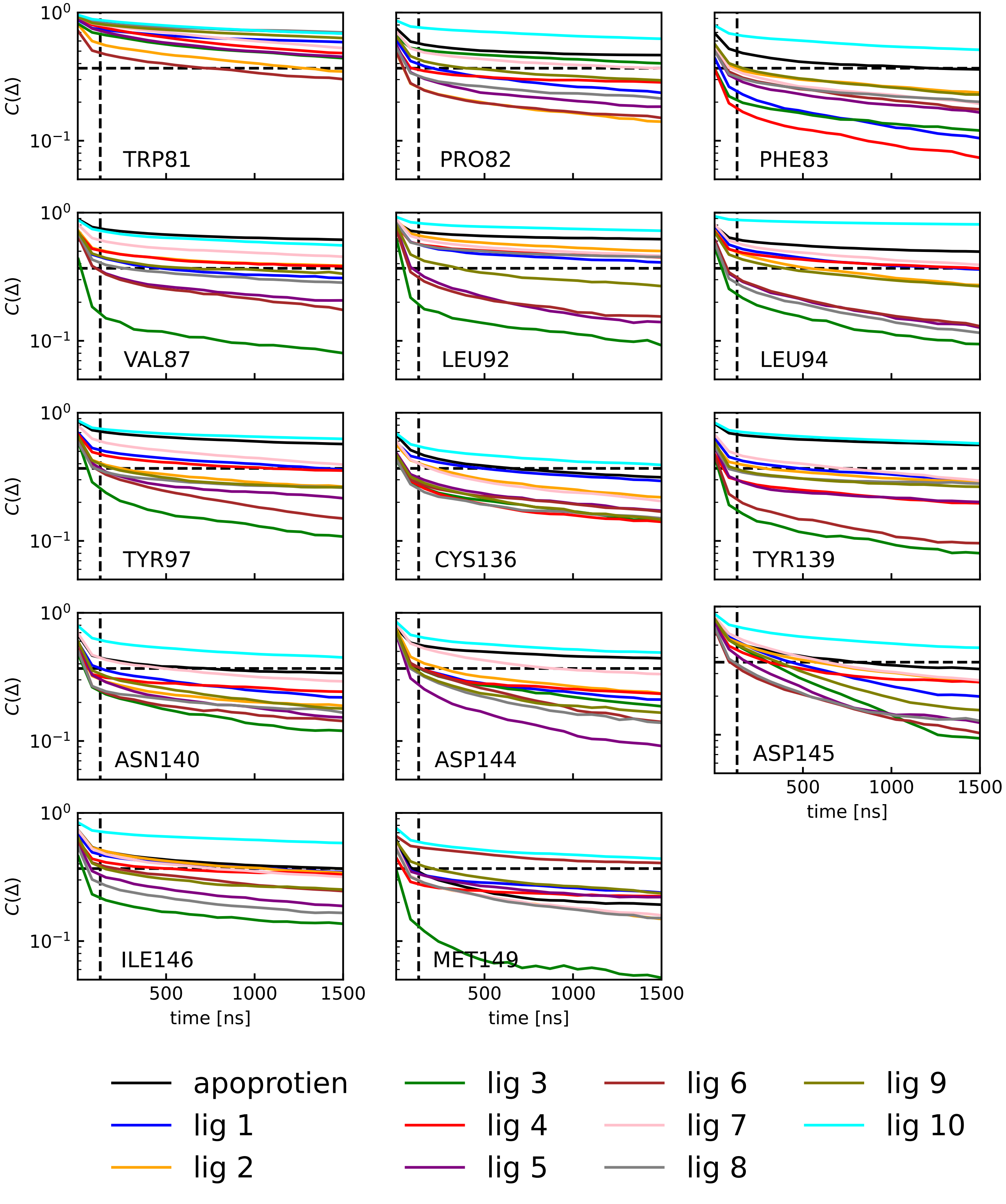

Supplementary Figure 7. Positional autocorrelation of 14 binding site residues in 11 systems (see Supplementary section *Analysis of fluctuation dynamics of the BRD4 systems* for details). Black dashed lines in x and y axes show x=128 ps and y=1/e, respectively.

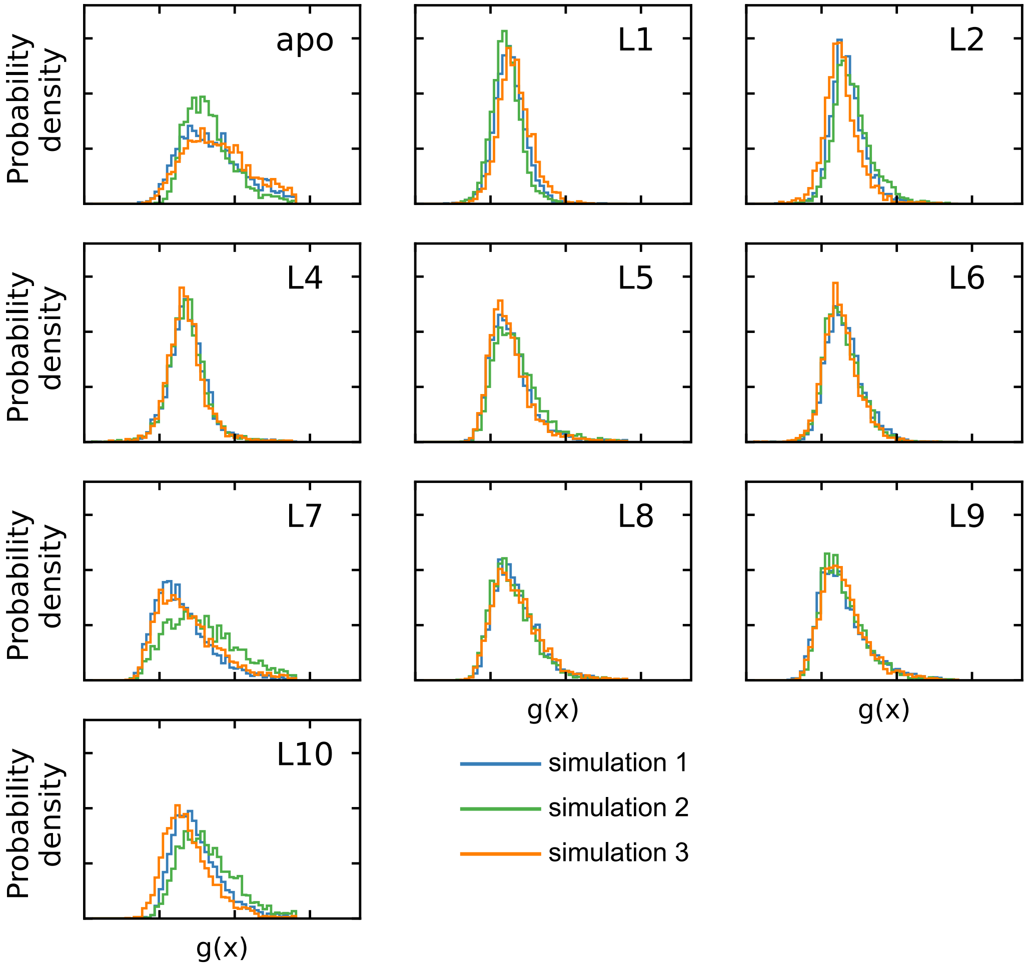

Supplementary Figure 8. Probability distributions of function $g(\boldsymbol{x})$ for each MD run in BRD4 systems (see Supplementary section *Evaluation of dynamics stability using* $g(x)$ for details). For the systems, the function $g(\boldsymbol{x})$ compared to system 3 was calculated.

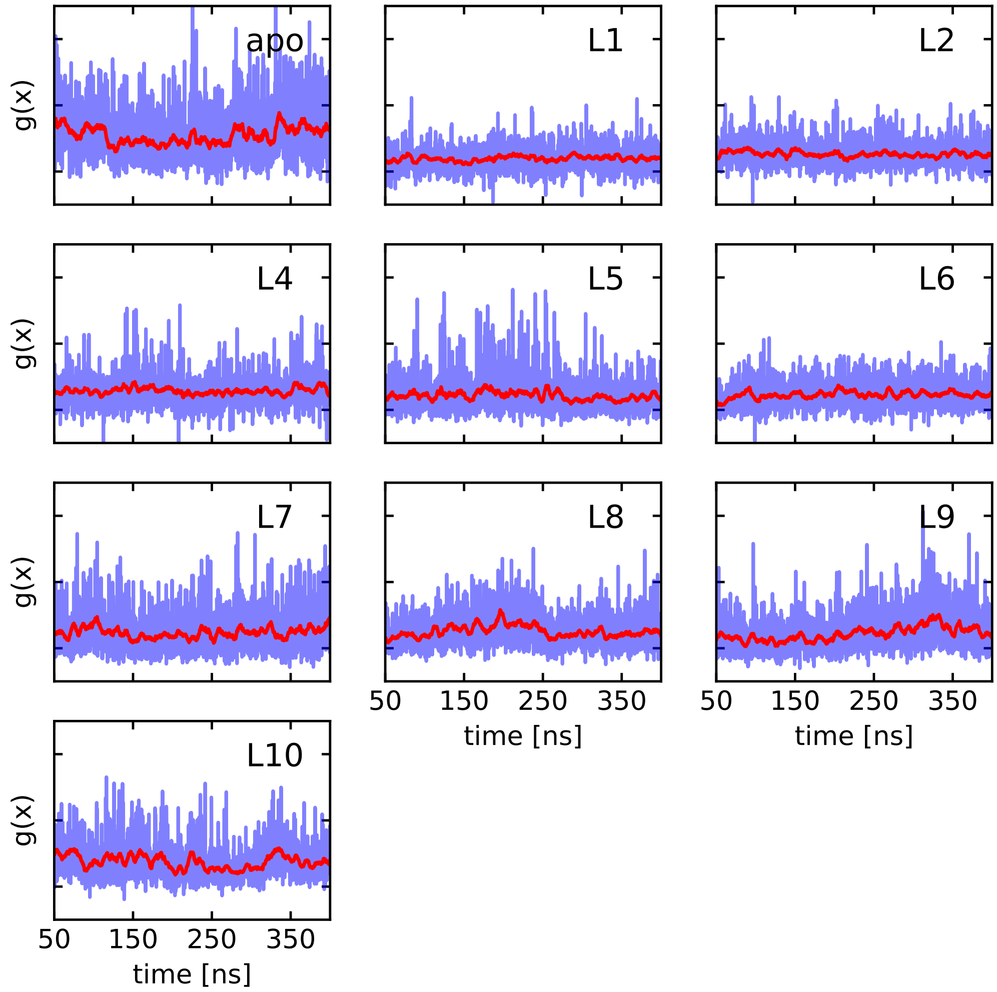

Supplementary Figure 9. Function $g(\boldsymbol{x})$ of simulation 1 of BRD4 systems is plotted to simulation time (see Supplementary section *Evaluation of dynamics stability using* $g(x)$ for details). For the systems, the function $g(\boldsymbol{x})$ compared to system 3 was calculated. Violet and red lines show the function $g(\boldsymbol{x})$ per 64 ps and moving average of the function $g(\boldsymbol{x})$ in 3.2 ns, respectively.

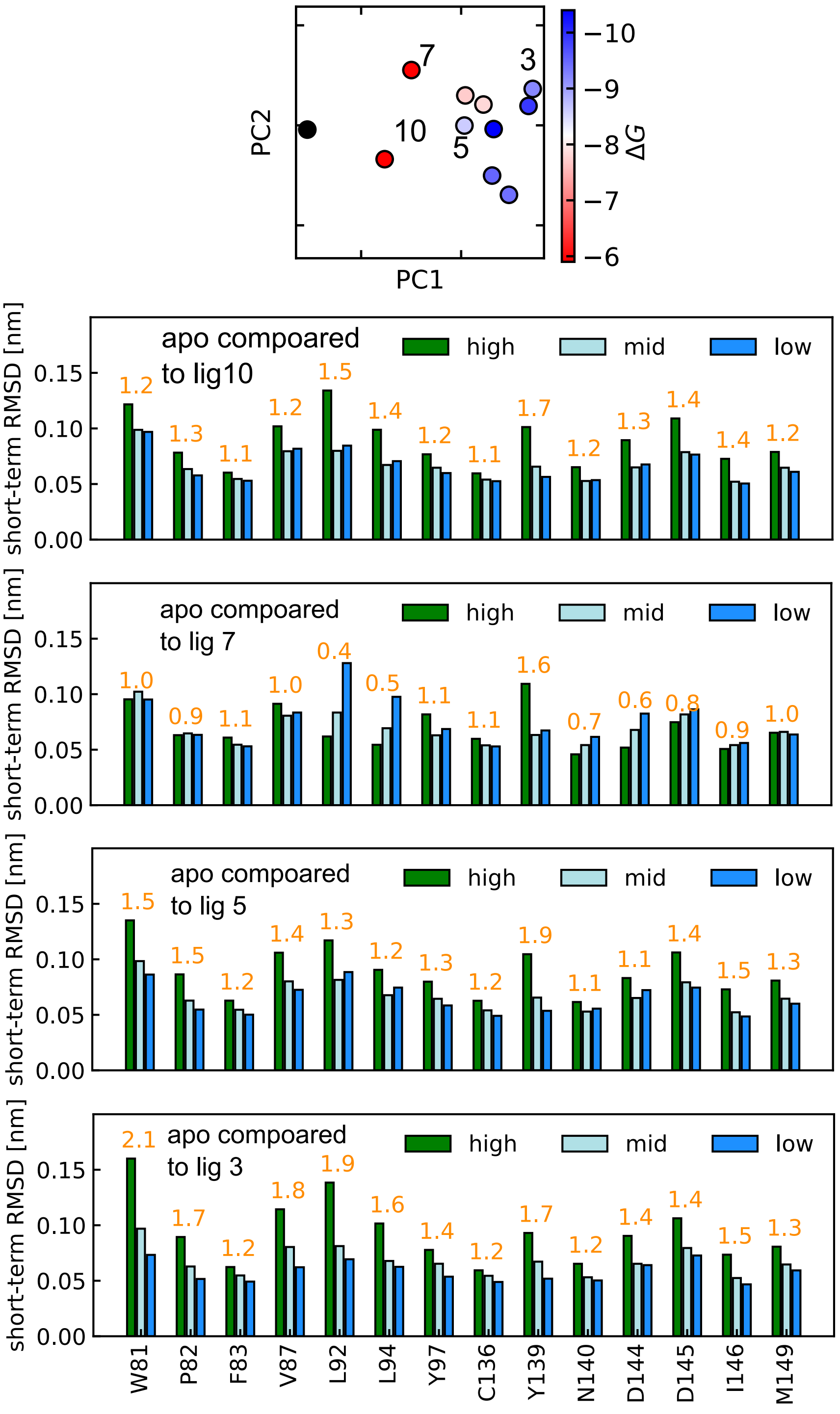

Supplementary Figure 10. Characteristic dynamics of BRD4 apoprotein, compared to holoprotein systems of increasing PC1 (system 3,5,7,10). Short-term RMSD was calculated for the holoprotein-like (low), apoprotein-like (high), and middle (mid) short-term dynamics. Orange number shows the ratio of the short-term RMSD of apoprotein-like dynamics to that of the holoprotein-like dynamics. The increasing PC1 mainly corresponds to lower mobility of W81.

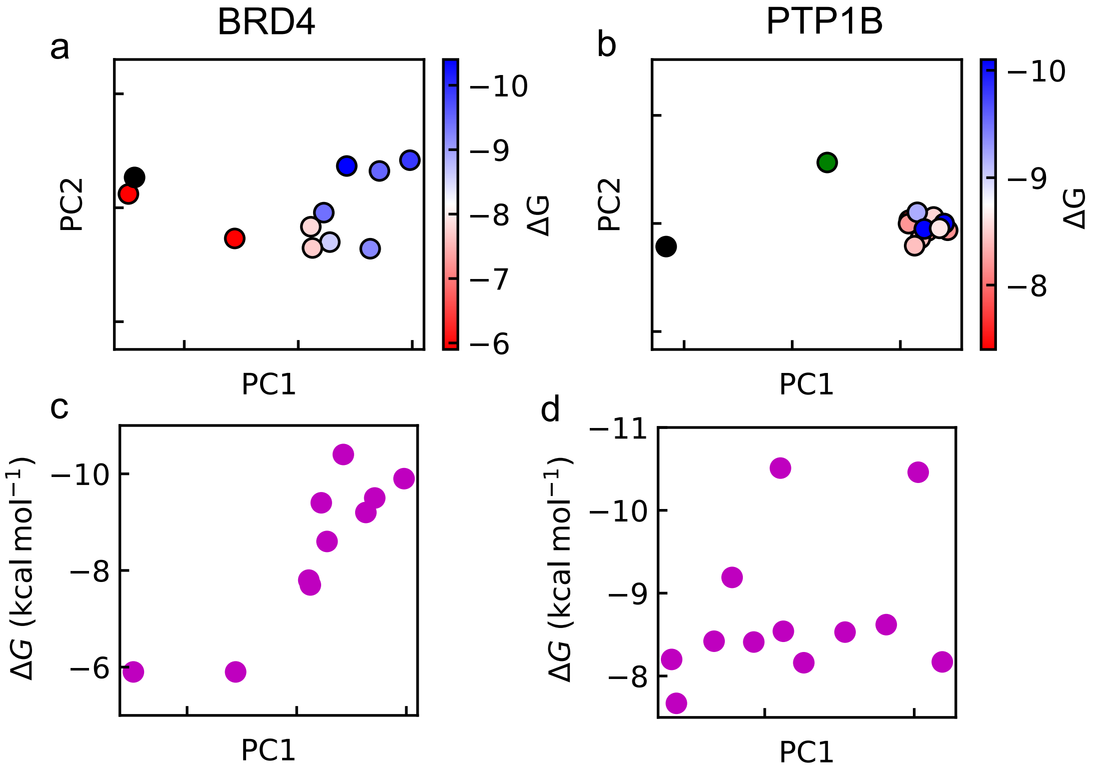

Supplementary Figure 11. Feature extraction using root mean square fluctuation (RMSF) at binding-site CAs and principal component analysis. The RMSF is calculated for each amino acid at the binding site in the last 150 ns of MD simulations (250–400 ns), and the RMSFs in three simulations for each system were averaged. Therefore, one system is represented by n features of the RMSF, where n is the number of the amino acids at the binding site. Subsequently, dimension reduction is performed using principal component (PC) analysis. (a) Map of PC1 and PC2 in BRD4 systems. (b) Map of PC1 and PC2 in PTP1B systems. (c) Correlation between the PC1 and the binding energies from free energy perturbation (FEP) in the BRD4 systems [5] (r=0.87). (d) Correlation between the PC1 and the binding energies from FEP in the PTP1B systems [6] (r=0.32).

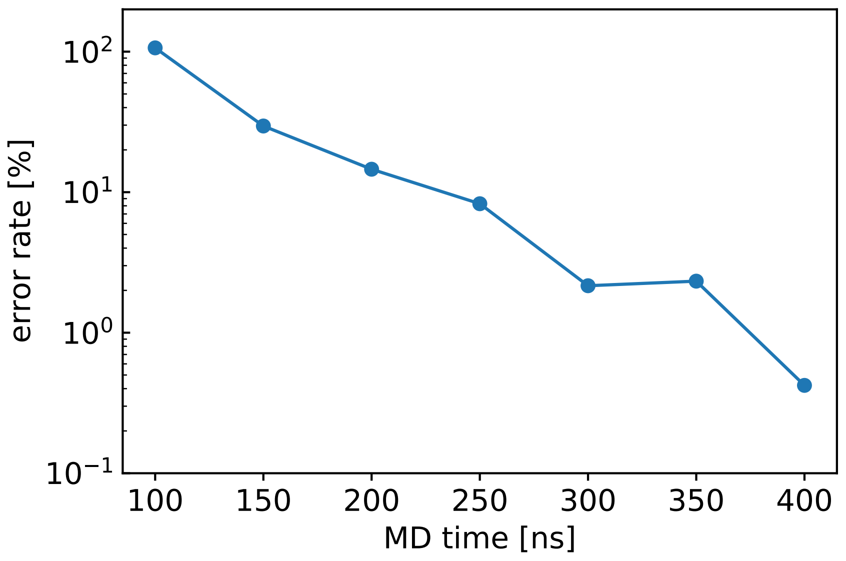

Supplementary Figure 12. Error ratio of Wasserstein distance calculated from shorter MD simulations of BRD4 systems (see Supplementary section *Molecular dynamics simulation length sufficient to cover LDE* for details.

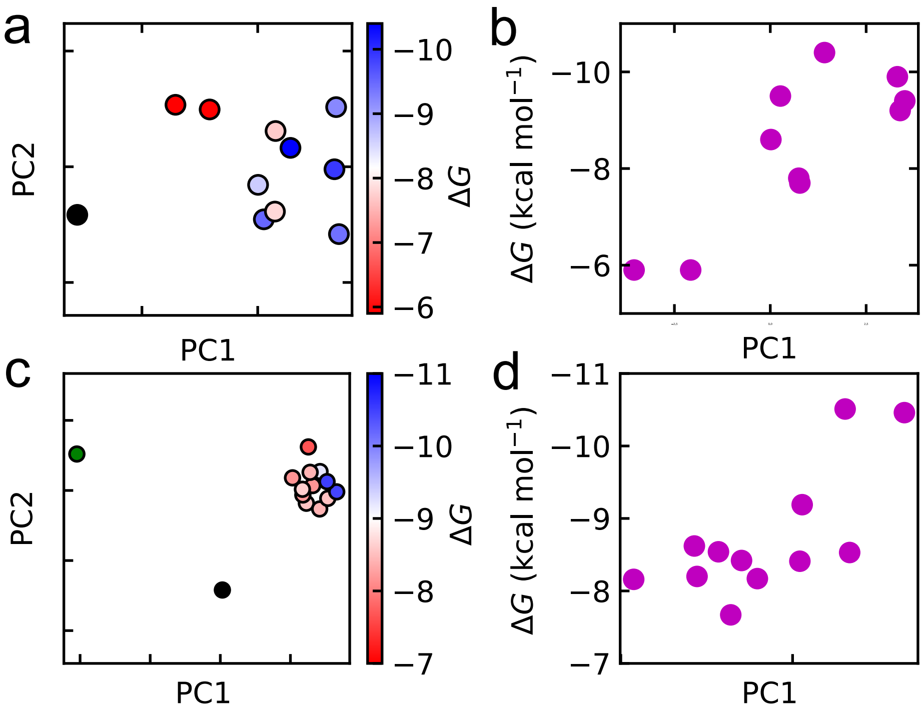

Supplementary Figure 13. Differences of LDE obtained from MD simulation in 50–200 ns. (a) Map of PC1 and PC2 in BRD4 systems. (b) Correlation between the PC1 and the binding energies from free energy perturbation (FEP) in the BRD4 systems [5] (r=0.81). (c) Map of PC1 and PC2 in PTP1B systems. (d) Correlation between the PC1 and the binding energies from FEP in the PTP1B systems [6] (r=0.72).

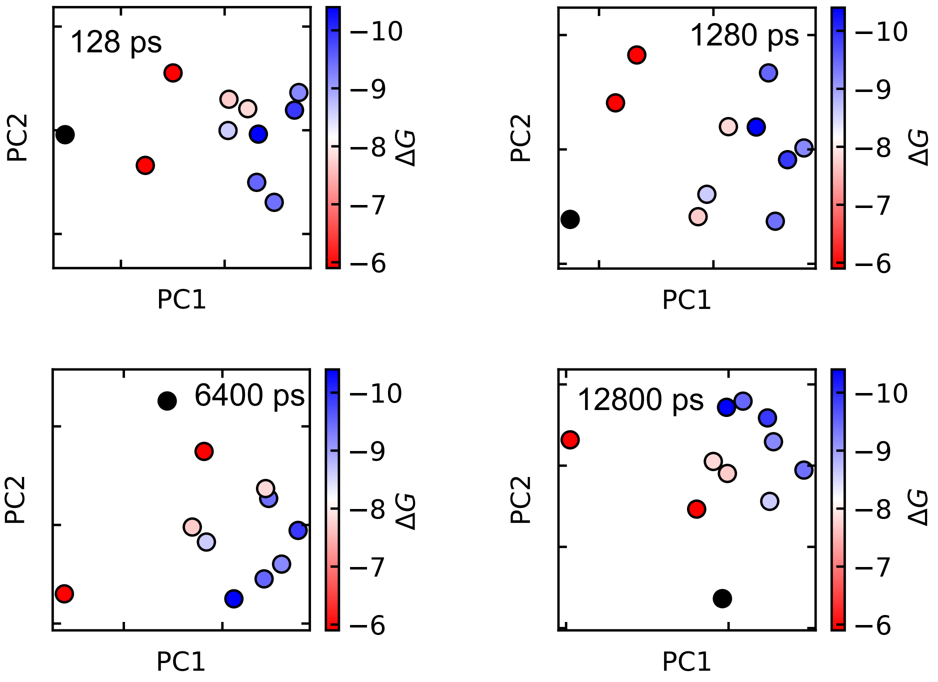

Supplementary Figure 14. Different time selections of LDE (128, 1,280, 6,400, and 12,800 ps) are compared for BRD4 systems (see Supplementary section *Selection of the local dynamics time length* for details.)

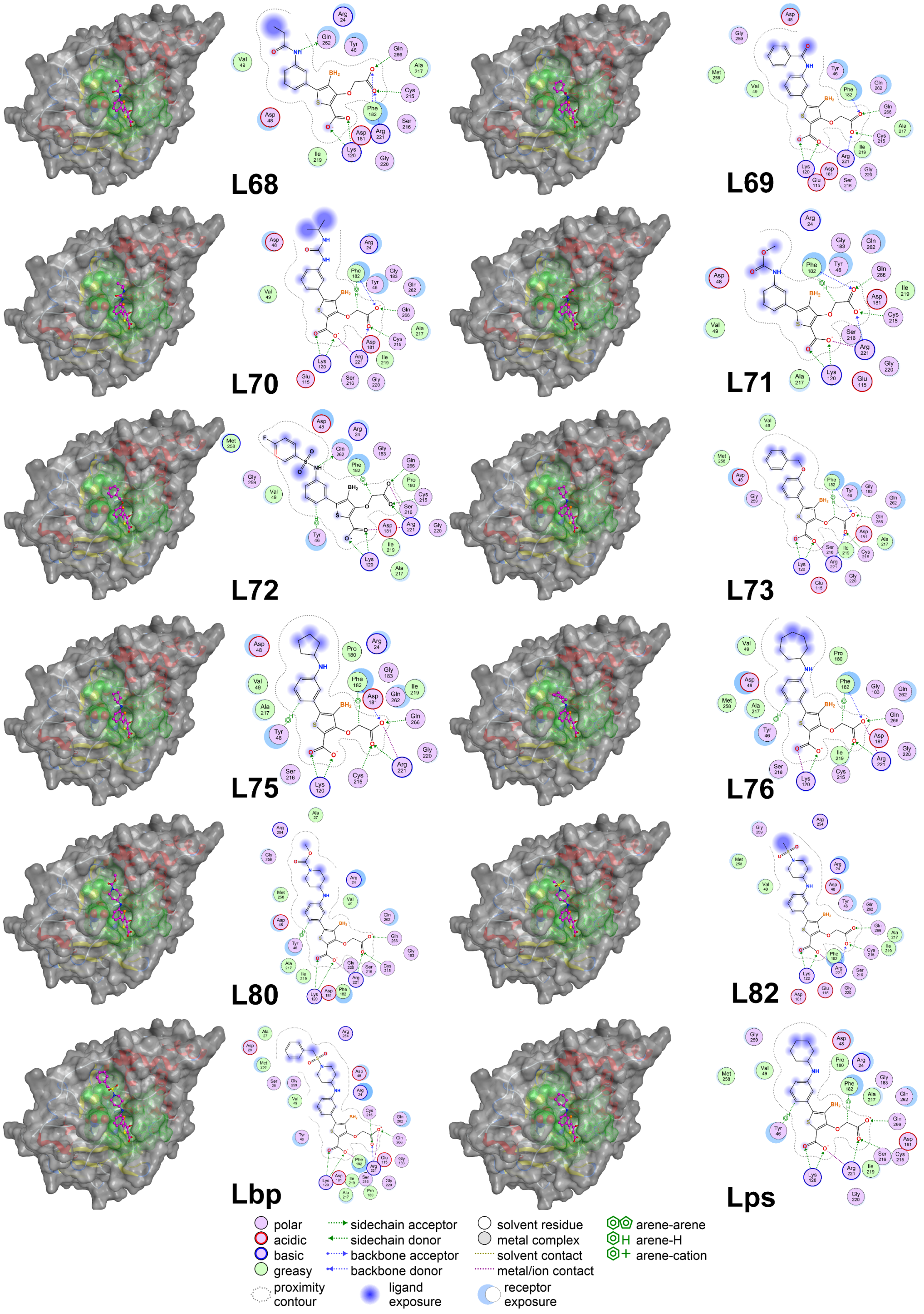

Supplementary Figure 15. PTP1B initial structures and 2D ligand interaction diagrams. Ribbon diagram of the configuration of PTP1B superimposed on the molecular surface. The ligand-binding site, residues in the ligand-binding site, and key residues are shown in green meshed molecular surfaces, pink balls and sticks, and green space-filling with labels, respectively. 2D diagrams are depicted by using the Molecular Operating Environment (MOE) software package [4]

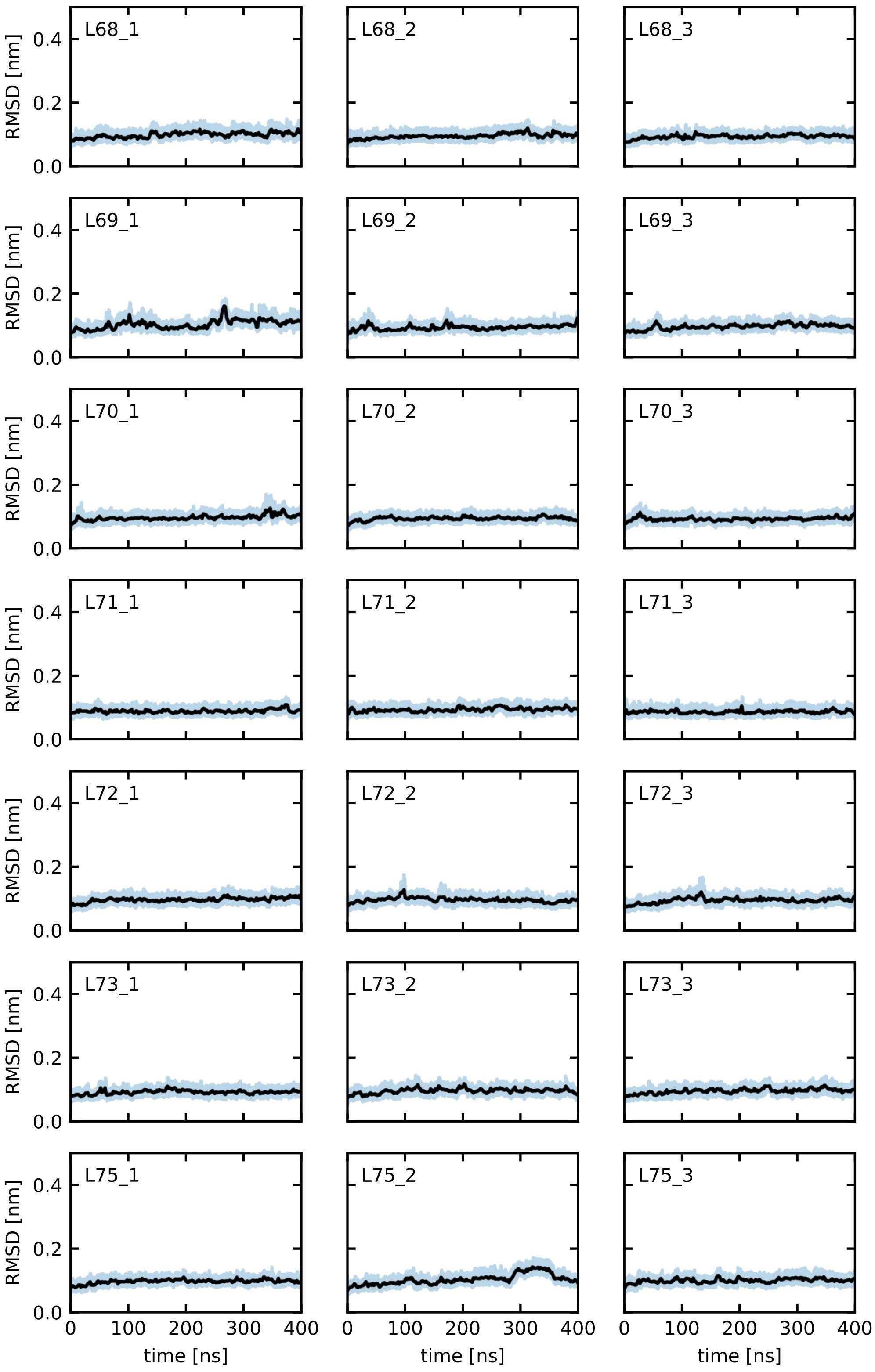
L68_1
L68_2
L68_3
L69_1
L69_2
L69_3
L70_1
L70_2
L70_3
L71_1
L71_2
L71_3
L72_1
L72_2
L72_3
L73_1
L73_2
L73_3
L75_1
L75_2
L75_3
RMSD [nm]
0.0
0.2
0.4
0
100
200
300
400
time [ns]

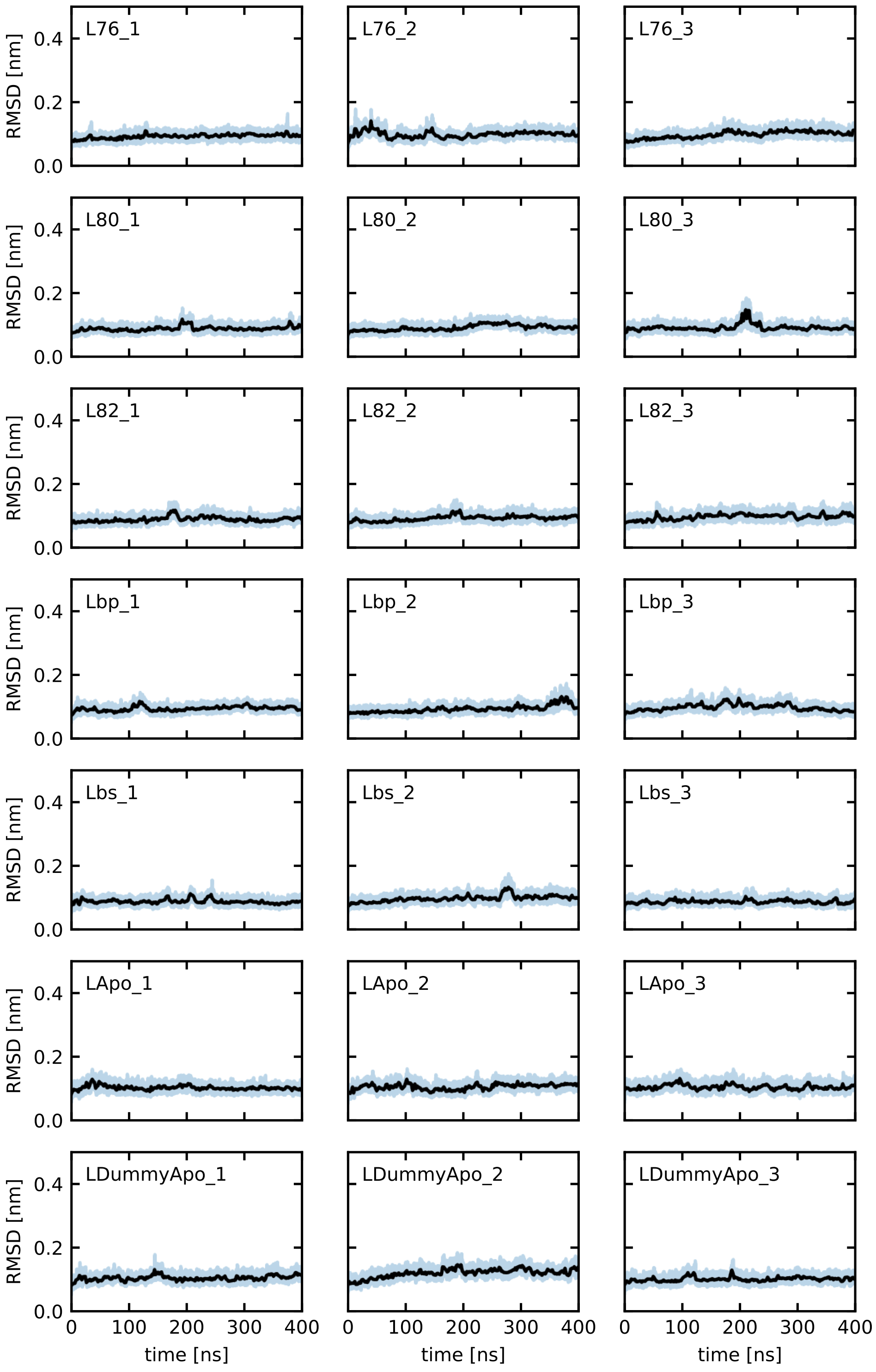

Supplementary Figure 16. RMS deviation of protein in PTP1B systems. CA atoms without terminals were used (2GLU-282MET). Structured were fitted by the CA atoms. Top-left label indicates system and simulation number. Light blue and black lines show the RMS deviation plotted in every 2 ps and moving average of the RMS deviation in 2 ns, respectively

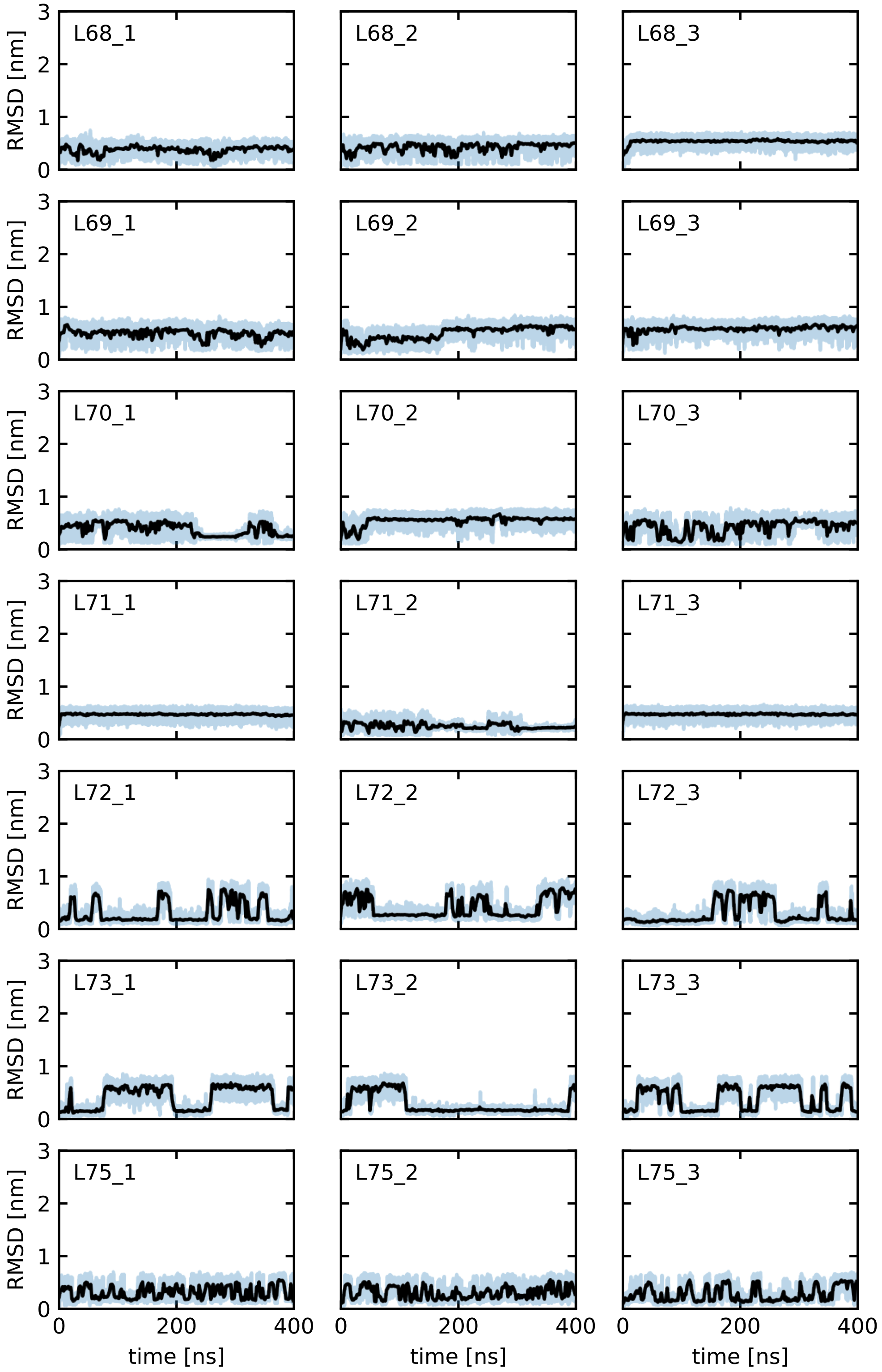
L68_1
L68_2
L68_3
L69_1
L69_2
L69_3
L70_1
L70_2
L70_3
L71_1
L71_2
L71_3
L72_1
L72_2
L72_3
L73_1
L73_2
L73_3
L75_1
L75_2
L75_3
RMSD [nm]
time [ns]

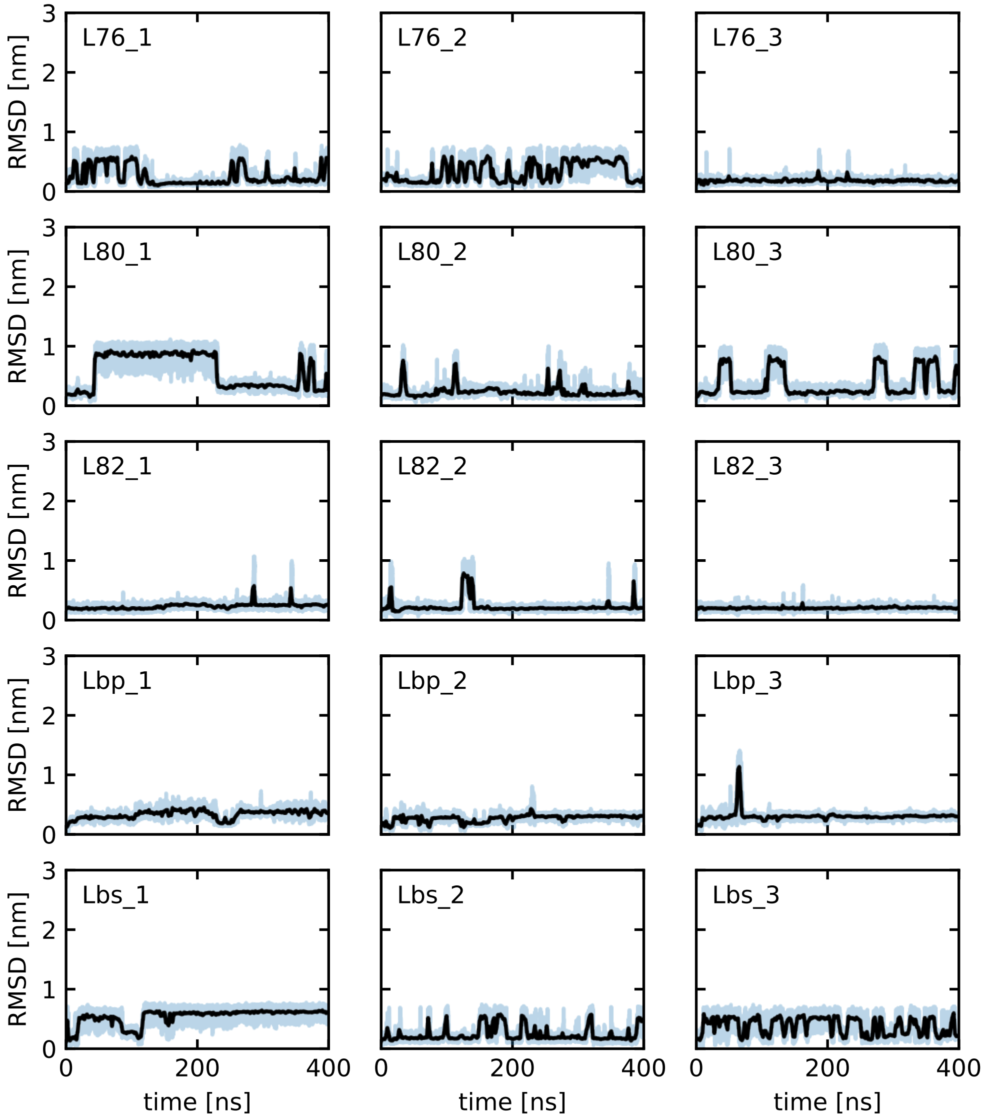

Supplementary Figure 17. RMS deviation of ligand in PTP1B systems. Translation and rotation were removed by fitting by protein CA atoms, and ligand heavy atoms were used to calculate the RMS deviation. Top-left label indicates system and simulation number. Light blue and black lines show the RMS deviation plotted in every 2 ps and moving average of the RMS deviation in 2 ns, respectively.

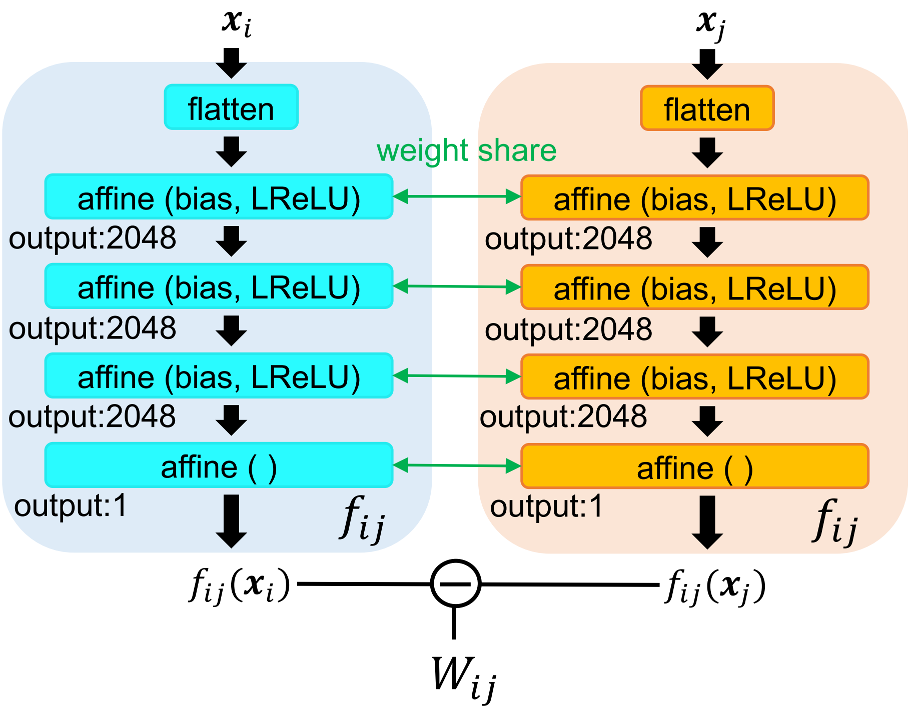

Supplementary Figure 18. Architecture of DNNs.

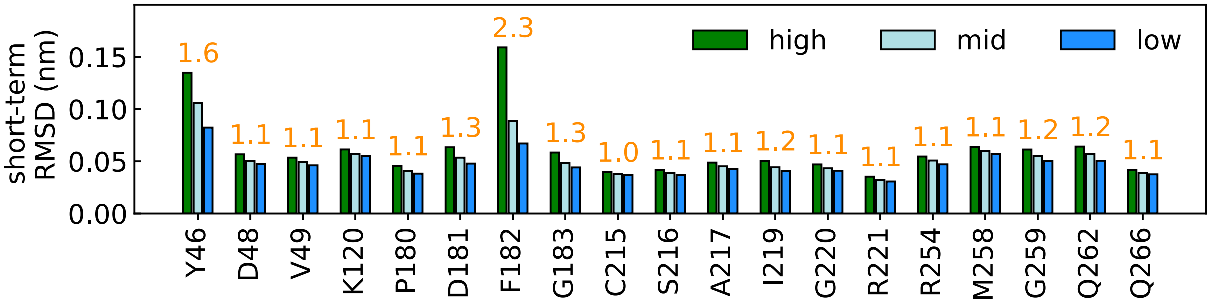

Supplementary Figure 19. Characteristic dynamics for residues in a PTP1B apoprotein system (LApo), in comparison to L82 system. The characteristic dynamics is observed in Try46 and Phe182.
